# Totally endoscopic aortic valve replacement: Techniques and early results

**DOI:** 10.3389/fcvm.2022.1106845

**Published:** 2023-01-09

**Authors:** Wenda Gu, Kan Zhou, Zhenzhong Wang, Xin Zang, Haijiang Guo, Qiang Gao, Yun Teng, Jian Liu, Biaochuan He, Huiming Guo, Huanlei Huang

**Affiliations:** ^1^Department of Cardiac Surgery, Guangdong Cardiovascular Institute, Guangdong Provincial People's Hospital, Guangdong Academy of Medical Sciences, Guangzhou, China; ^2^The Second School of Clinical Medicine, Southern Medical University, Guangzhou, China

**Keywords:** total endoscopic, minimally invasive, aortic valve replacement, standard prosthesis, endoscopic cardiac surgery

## Abstract

**Objective:**

To demonstrate the technical details of total endoscopic aortic valve replacement using a standard prosthesis, compare the clinical effect and safety of endoscopic aortic valve replacement and traditional aortic valve replacement.

**Methods:**

From 2020 to 2021, 60 consecutive patients underwent elective isolated aortic valve replacement (AVR). They were divided into two groups: the total endoscopic AVR group (TE-AVR group, 29 patients, nine women, aged 51.65 ± 11.79 years), and the traditional full-sternotomy group (AVR group, 31 patients, 13 women, aged 54.23 ± 12.06 years). Three working ports were adopted in the TE-AVR procedure.

**Results:**

No patient died in either group. The cardiopulmonary bypass (CPB) time and aortic cross-clamp (ACC) time in the TE-AVR group were longer than those in the AVR group (CPB time: 177.6 ± 43.2 vs. 112.1 ± 18.1 min, *p* < 0.001; ACC time: 118.3 ± 29.7 vs. 67.0 ± 13.2 min, *p* < 0.001). However, the mechanical ventilation duration (14.2 ± 9.3 vs. 24.0 ± 18.9 h, *p* = 0.015) and postoperative hospital stay (6.0 ± 1.7 vs. 8.0 ± 4.5 days, *p* = 0.025) were shorter in patients of TE-AVR group than those of AVR group. Although the ICU stay (55.1 ± 26.9 vs. 61.5 ± 44.8 h, *p* = 0.509) and post-operative chest drainage of the first 24 h (229.8 ± 125.0 vs. 273.2 ± 103.2 ml, *p* = 0.146) revealed no statistical difference, there was a decreasing trend in the TE-AVR group. Among the patients of the TE-AVR group, two patients were converted to thoracotomy because of mild to moderate paravalvular leakage identified by intraoperative transesophageal echocardiography.

**Conclusion:**

Total endoscopic aortic valve replacement is safe and feasible, with less trauma and quicker recovery.

## 1. Introduction

After decades of development, aortic valve replacement (AVR) *via* traditional median thoracotomy has been proven to be a safe and effective procedure with low mortality and morbidity. In recent years, patients and surgeons have been seeking better minimally invasive aortic valve surgery methods due to increasing demand for aesthetics, less trauma, and rapid recovery. The current minimally invasive surgical procedures for the aortic valve include transcatheter aortic valve implantation (TAVI), partial sternotomy AVR, right anterolateral thoracotomy AVR, right parasternal transverse thoracotomy AVR, right parasternal longitudinal thoracotomy AVR, and total endoscopic surgery. Over the past decade, TAVI has gradually emerged as an effective alternative to surgery in high or intermediate-risk patients and has entered the guidelines for aortic valve surgery (1, 2). However, it is still controversial whether TAVI is optimal for low-risk and intermediate-risk patients due to relatively high incidences of perioperative cerebral complications, permanent pacemaker implantation, and paravalvular leakage, as well as the potential disadvantage of the preservation of aortic valve leaflet tissue (3, 4).

With the rapid development of surgical techniques and operating instruments, total endoscopic surgery through the right chest wall approach has been widely used in repairing atrial septal and ventricular septal defects, mitral valve repair, or replacement surgery (5, 6). However, AVR surgeries using total endoscopy are rarely reported, which may be related to the particularity of the anatomy of the aortic root. The aortic root has a small space, and it isn't easy to operate on the severely calcified tissue, affecting total endoscopic AVR (TE-AVR) performance. Therefore, this study aimed to investigate the safety and feasibility of total endoscopic aortic valve replacement and compare AVR's clinical effects *via* total endoscopy and traditional median thoracotomy.

## 2. Methods

### 2.1. Patient selection

This study was proved by the ethics review committee of Guangdong Provincial People's Hospital (XJS2021-025-02). Data were collected from our center's valve and coronary surgery database. In this study, we screened 60 patients with aortic valve disease between 2020 and 2021, of whom 29 met the selection criteria for total endoscopic aortic valve replacement (TE-AVR group). During the same period, 31 patients who met the requirements for total endoscopic replacement but chose conventional surgery were enrolled as a control group (AVR group).

The inclusion criteria for TE-AVR in our center were as follows: (1) patients with stand-alone aortic valve disease; (2) diameter of ascending aorta <45 mm; (3) diameter of aortic root >25 mm, and diameter of aortic annulus >20 mm.

The exclusion criteria for TE-AVR included: (1) thoracic deformity and a history of right thoracic surgery. (2) bilateral femoral artery diseases or aortic malformation, severe aortic atherosclerosis, or other cardiac malformations (patent ductus arteriosus, persistent left superior vena cava); (3) severe pulmonary hypertension.

Data of patients undergoing AVR through total endoscopic surgery and aortic valve replacement through median thoracotomy were statistically analyzed. The comparison data included in-hospital mortality, duration of mechanical ventilation, time of intensive care unit (ICU) stay, postoperative hospital stay, blood transfusion rate, thoracic drainage, and postoperative complications, including low cardiac output syndrome, respiratory failure, stroke, myocardial infarction, paravalvular leakage, reoperation due to bleeding and wound infection.

### 2.2. Surgical technique

#### 2.2.1. Total endoscopic surgery

**Figure 1:**
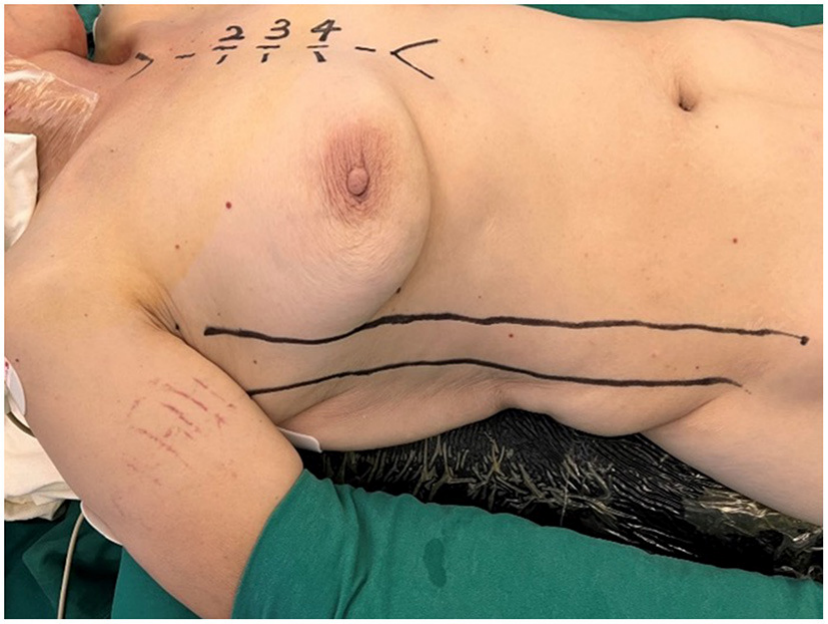
The patient was positioned supine with the right hemithorax elevated 20–30 degrees, the right upper limb was placed parallel to the body, and the elbow joint was slightly flexed.

The patient was positioned supine with the right hemithorax elevated 20–30 degrees, the right upper limb was placed parallel to the body, the elbow joint was slightly flexed, and the right anterior lateral wall of the chest was fully exposed (see Figure 1). Double-lumen or single-lumen tracheal intubation was conducted under general anesthesia. Femoral artery cannulation (18 or 20 Fr) and femoral vein cannulation (28 Fr) were deployed to establish peripheral cardiopulmonary bypass (CPB). It was unnecessary to cannulate the superior vena cava, the femoral vein cannulation should be one size larger than the conventional bi-cava cannulation, and the vacuum was compulsory for adequate venous drainage.

**Figure 2:**
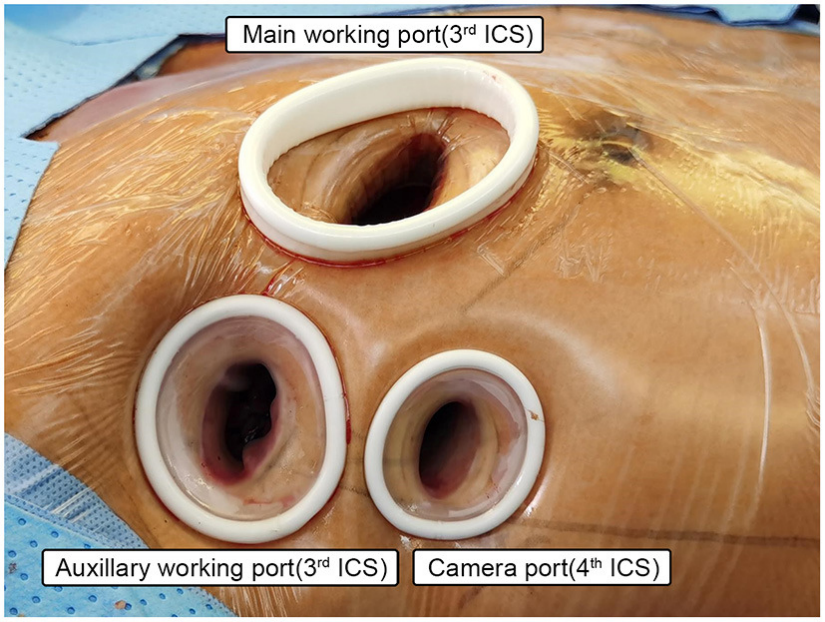
Setup of the working ports. **Main working port:** 3–4 cm in length, for patients with aortic valve insufficiency, the main working port was located between the midclavian line and anterior axillary line of the right 3rd intercostal space (ICS); in patients with aortic valve stenosis (AS), the main operating port was located between the parasternal line and the midclavicular line of the right 3rd ICS; mainly used for primary surgical instruments and delivery of cardioplegia. **Auxiliary working port:** 1.5–2 cm in length, located between the anterior axillary line and the midaxillary line of the 3rd ICS, mainly used for placing a CHITWOOD aortic cross-clamp, a catheter for left ventricular venting, and instruments operated by the surgeon's left hand. **Camera port:** 1–1.5 cm in length, located between the 4th ICS between the anterior axillary line and the midaxillary line.

Three working ports were adopted. The position of the main operating port differed according to the type of aortic valve disease: in patients with aortic valve insufficiency, the main working port was located between the midclavian line and anterior axillary line of the right 3rd intercostal space (ICS); in patients with aortic valve stenosis (AS), the main operating port was located between the parasternal line and the midclavicular line of the right 3rd ICS; the main working port was 3–4 cm in length, mainly used for primary surgical instruments and delivery of cardioplegia. The second operation port (auxiliary port) was located between the anterior axillary line and the midaxillary line of the 3rd ICS, with a length of 1.5–2 cm. It was mainly used for placing a CHITWOOD aortic cross-clamp, a catheter for left ventricular venting, and instruments operated by the surgeon's left hand (such as grasping forceps). The camera port was located between the 4th ICS between the anterior axillary line and the midaxillary line, with a 1–1.5 cm length. Usually, the camera port was about 0.5 cm lower than the auxiliary port. It was closer to the level of the midaxillary line for less interference between the endoscopy and surgical instruments (see Figure 2).

Immediately after each incision, a soft tissue retractor was placed to protect the subcutaneous tissue and muscle. The pericardium was incised in parallel at 1–2 cm above the phrenic nerve, suspended and fixed with force on the ipsilateral side of the operator, which was helpful for better exposure of the aortic root. 3–0 prolene suture with pledget is used as a purse for a cardioplegia catheter. Patients with AS without or only mild regurgitation can insert the catheter and remove it after cardioplegia delivery. And for patients with the principal diagnosis of aortic regurgitation (AR), only the purse-string suture was placed, and no cardioplegia catheter was inserted. In addition, a 5–0 prolene stitch with a pledget was placed on the central part of the right atrial appendage near the aorta and retracted and fixed to the diaphragm surface. Thus excellent exposure of the aortic root might be achieved. The CHITWOOD cross-clamp was placed through the auxiliary port. As for the administration of cardioplegia, Del-Nido and HTK cardioplegia solutions were more favorable for longer re-dosing intervals. The patients with AS could be anterogradely administrated through the catheter. And for patients with AR, the cardioplegia was delivered through coronary Ostia directly.

After delivery of cardioplegia, the aortic incision was extended toward the right/left commissural on the left side and to the non-coronary sinus near the left/non-commissural on the right side. A stitch was placed at the center of the superior edge of the aortic incision to pull it to the upper right and fix it on the lower edge of the pericardium. Two stitches were placed at the inferior edge of the aortic incision. These stitches were pulled and settled on the upper edge of the pericardium and the diaphragm surface. Then the aortic valve and valve annulus can be completely exposed, and if necessary, additional stitches can be placed at the left/right commissural for traction. After complete exposure of the aortic valve, resection of aortic leaflets and placement of the stitches of the prosthesis was the same as for traditional AVR surgery. A 2–0 double-ended needle polyester stitch with a pledget was used for intermittent mattress sutures in our center. The pledgets could be positioned above or below the aortic valve annulus.

**Figure 3:**
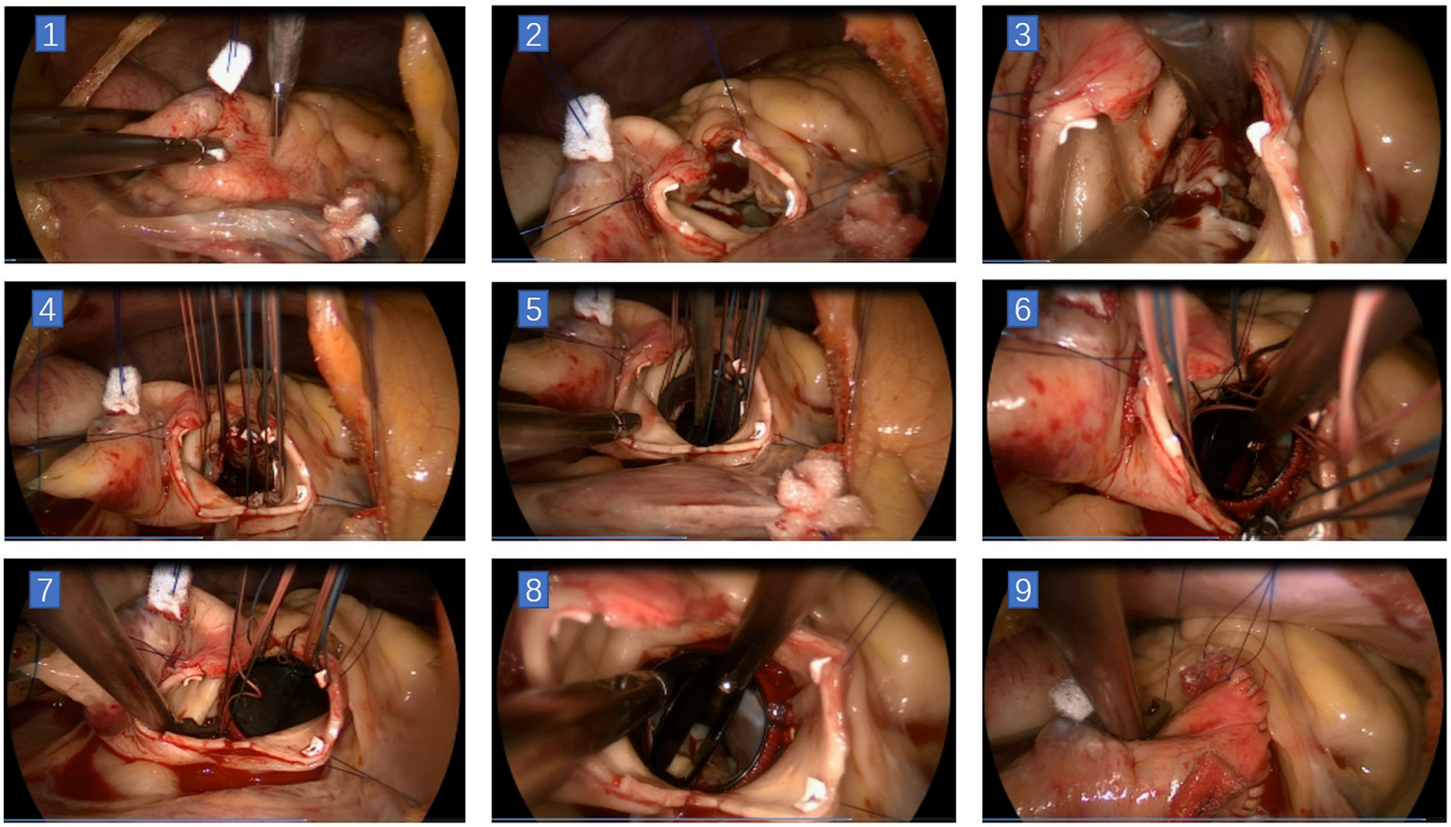
Main steps of total endoscopic aortic valve replacement. **(1)** Incision on ascending aorta. **(2)** The aortic incision was extended toward the right/left commissural on the left side and to the non-coronary sinus near the left/non-commissural on the right side. A stitch was placed at the center of the superior edge, and two stitches were placed at the inferior edge of the aortic incision. **(3)** Resection of the aortic leaflets. **(4)** 2–0 polyester stitches with pledges were used for intermittent sutures of the prosthesis. **(5)** Seating the prosthesis valve. **(6)** Knotting could be done in the order of first non-coronary annulus, then left coronary annulus, and finally right coronary annulus with a unique minimally invasive knot pusher. **(7)** Remove the stitches in the same previous order. **(8)** Check the status of the prosthesis. **(9)** Close the aortic incision.

We performed in the order of suturing the right coronary annulus first, then the left coronary annulus, and finally the non-coronary annulus. Before placing non-coronary annulus stitches, the sizer was used to determine the size of the prosthetic valve. Knotting could be done in the order of first non-coronary annulus, then left coronary annulus, and finally right coronary annulus with a unique minimally invasive knot pusher (some can also be knotted directly by hand). After the aortic incision was closed, a needle was inserted at the root for de-airing, then the cross-clamp was removed. Each patient underwent transesophageal echocardiography while dismantling the CPB (see Figure 3).

#### 2.2.2. Traditional median thoracotomy

The patient was positioned supine, single-lumen tracheal tube was intubated. The sternum was sawed through a traditional midline incision. CPB was routinely established through ascending aorta cannulation, the one-stage cavo-atrial cannulation, and the right upper pulmonary vein drainage. After sufficient cooling and cross-clamping of the aorta, aortic valve replacement was routinely completed through an oblique incision at the aortic root. After the aortic incision was sutured, a needle was inserted at the aorta root for de-airing. Each patient underwent transesophageal echocardiography while dismantling the CPB.

### 2.3. Statistical analyses

All statistical analyses were conducted using IBM SPSS Statistics for macOS, Version 25.0 (Released 2017, IBM Corporation, Armonk, NY, USA). Data in normal distribution were expressed as mean ± standard deviation, and the independent sample *t*-test was used for the analysis of the two groups; the categorical count data were expressed as the number of cases (n) or percentage (%), and the two groups were analyzed by the chi-square test or Fisher's exact test. The difference was considered statistically significant at *p* < *0.05*.

## 3. Results

### 3.1. Baseline characteristics

The present study consisted of 29 patients in the TE-AVR group and 31 in the AVR group. In the TE-AVR group, there were 20 males (69.0%) and nine females (31.0%), with an average age of (51.7 ± 11.8) years, an average weight of (64.7 ± 9.6) kg, left ventricular ejection fraction (LVEF) (61.0 ± 7.9) %, the diameter of ascending aorta (AAo) (37.6 ± 3.4) mm, left ventricular end-diastolic diameter LVEDD (58.8 ± 10.1) mm, including 4 cases of smoking and 13 cases of hypertension. Among the 31 cases of the AVR group, there were 18 males (58.1%) and 13 females (41.9%), with average age (54.2 ± 12.1) years, average weight (63.0 ± 11.7) kg, LVEF (56.1 ± 11.6) %, the diameter of AAo (36.0 ± 5.3) mm, LVEDD (46.9 ± 7.5) mm, including 3 cases of smoking, 5 cases of hypertension, 1 case of diabetes, and 1 case of chronic obstructive pulmonary disease (COPD). There were no significant differences in gender, age, body weight, LVEF, the diameter of ascending aorta, and underlying conditions (except hypertension) between the two groups (*p* > 0.05), but the LVEDD in the TE-AVR group was significantly greater than that in the AVR group (Table 1).

**Table 1:** Preoperative baseline data.

**Variables**	**TE-AVR (*n* = 29)**	**AVR (*n* = 31)**	***P-*value**
Female, *n* (%)	9 (31.0)	13 (41.9)	0.381
Age	51.7 ± 11.8	54.2 ± 12.1	0.408
Body weight, kg	64.7 ± 9.6	63.0 ± 11.7	0.539
LVEF, %	61.0 ± 7.9	56.1 ± 11.6	0.065
Diameter of AAo, mm	37.56 ± 3.4	36.0 ± 5.3	0.187
LVEDD, mm	58.8 ± 10.1	46.9 ± 7.5	0.000
Smoking, *n* (%)	4 (13.8)	3 (9.6)	0.702
**Comorbidities**			
Hypertension, *n* (%)	13 (51.7)	5 (16.2)	0.015
Diabetes, *n* (%)	0 (0)	1 (3.2)	1.000
COPD, *n* (%)	0 (0)	1 (3.2)	1.000

Values are presented as mean ± standard deviation or n (%). The differences between the two groups were analyzed by t-test for continuous variables and X^2^ or Fisher exact tests for categorical variables. The statistical significance was set as p < 0.05. TE-AVR, total endoscopic aortic valve replacement; AVR, aortic valve replacement; LVEF, left ventricular ejection fraction; AAo, ascending aorta; LVEDD, left ventricular end-diastolic diameter; COPD, chronic obstructive pulmonary disease.

### 3.2. Intraoperative and postoperative results

In the TE-AVR group, 20 mechanical valves and nine prostheses were implanted; one patient underwent aortic root enlargement (Nick's procedure), and one underwent valve annulus reconstruction and aortic wall repair in one case. There was no operative death, no re-thoracotomy for hemostasis, and no low cardiac output and stroke occurred in all patients. Intraoperative transesophageal echocardiogram (TEE) showed mild to moderate paravalvular leakage of the aortic valve in 2 cases, both of which were mechanical valves. Considering the difficulty of repair under endoscopy, these two cases were converted to sternotomy. Then the aorta was re-clamped, and the paravalvular leak was repaired.

The CPB and aortic cross-clamping (ACC) times in the TE-AVR group were longer than those in the AVR group (CPB time: 177.7 ± 43.2 vs. 112.1 ± 18.1 min, *p* < 0.001; ACC time: 118.3 ± 29.7 vs. 67.0 ± 13.2 min, *p* < 0.001). But the time of mechanical ventilation (14.2 ± 9.3 vs. 24.0 ± 18.9 h, *p* = 0.015) and postoperative hospital stay (6.0 ± 1.7 vs. 8.0 ± 4.5 days, *p* = 0.025) were shorter than those of AVR group. Compared with the AVR group, for ICU stay (55.1 ± 26.9 vs. 61.5 ± 44.8 h, *p* = 0.509) and chest drainage of the first 24 h (229.8 ± 125.0 vs. 273.2 ± 103.2 ml, *p* = 0.146), although not statistically different, there were decreasing trends in the TE-AVR group (Table 2).

**Table 2:** Perioperative characteristics.

**Variables**	**TE-AVR (*n* = 29)**	**AVR (*n* = 31)**	***P-*value**
CPB time, minutes	177.7 ± 43.2	112.1 ± 18.1	**<** **0.001**
ACC time, minutes	118.3 ± 29.7	67.0 ± 13.2	**<** **0.001**
Mechanical ventilation time, hours	14.2 ± 9.3	24.0 ± 18.9	**0.015**
ICU stay, hours	55.1 ± 26.9	61.5 ± 44.8	0.509
Postoperative hospital stay, days	6.0 ± 1.7	8.0 ± 4.5	**0.025**
Chest drainage of the first 24 h, ml	229.8 ± 125.0	273.2 ± 103.2	0.146
In-hospital deaths, *n* (%)	0 (0)	0 (0)	1.000
**Complications**, ***n*** **(%)**			
Paravalvular leakage	2 (6.9)	0 (0)	0.229
Re-operation	0 (0)	0 (0)	1.000
Stroke	0 (0)	0 (0)	1.000

Values are presented as mean ± standard deviation or n (%). The differences between the two groups were analyzed by t-test for continuous variables and X^2^ or Fisher exact tests for categorical variables. The statistical significance was set as p < 0.05. TE-AVR, total endoscopic aortic valve replacement; AVR, aortic valve replacement; CPB, cardiopulmonary bypass; ACC, aortic cross-clamp; ICU, intensive care unit.

## 4. Discussion

Aortic valve disease, including aortic insufficiency or aortic stenosis, is one of the most common structural cardiac diseases. Since the first AVR was performed through median thoracotomy in 1964, it has been regarded as one of the most classical cardiopulmonary bypass cardiac surgeries. The surgical exposure is excellent, and the surgeons can operate conveniently through median sternotomy. However, the shortcomings of sternotomy are also apparent. Not only is there much traumatic bleeding, but it also destroys the integrity of the sternum, which increases the rate of poor wound healing. For patients with osteoporosis, especially the elderly, the sternum is easily cut by the steel wire, causing the sternum to be unhealing, thus affecting postoperative recovery (7).

In recent years, surgeons have made many attempts in terms of surgical strategies in minimally invasive aortic valve surgery to reduce patients' physical and mental trauma and meet the needs of young patients for aesthetics. In 1996, Konertz et al. first reported the AVR through the small incision of the upper sternum (8), many cardiac surgeons tried one after another, and the shape of the sternum incision gradually evolved from the “T” and “V” shape to the classic “J” shape or reverse. The “L” shape has become the most used minimally invasive AVR surgery (9). However, although these surgeries avoid full incision of the sternum, they still destroy the stability of the sternum. Nevertheless, converting to total sternotomy from this type of small incision may increase mortality and complication rates: in 2007, Tabata et al. found that mortality and complication rates were 2.6 and 4.0%, respectively, in patients who were converted to total sternotomy from the upper or lower mini-sternotomy, and the main reasons were bleeding, inadequate exposure of the operative field, and left ventricular dysfunction (10). In 1996, Cosgrove et al. first reported that 25 patients underwent AVR through the right parasternal longitudinal incision (11). Still, the incision extended from the lower edge of the second costal cartilage to the upper edge of the fifth costal cartilage. The 3rd and 4th costal cartilages were resected, and although the sternum was intact, the trauma was still significant. In 1997, Benetti et al. reported for the first time AVR through the right anterolateral ICS incision (6–8 cm) and, at the same time, through the femoral artery and the right atrium cannulas to establish extracorporeal circulation (12). AVR through a parasternal transverse incision (5–7 cm) has also been reported. This approach avoided splitting the sternum and maintained the integrity of the thoracic cage, which laid a solid foundation for the subsequent endoscopic-assisted and total endoscopic AVR. There were also certain limitations of the parasternal transverse incision. A rib retractor was necessary, which might be a potential risk of damaging the internal mammary artery. And CT examination was mandatory before surgery. A parasternal transverse incision would not be suitable if the ascending aorta were significantly leftward. Although these minimally invasive procedures have limitations, many studies have shown that minimally invasive aortic valve surgery has less bleeding, faster recovery, fewer postoperative complications, and comparable long-term survival rates compared to traditional median thoracotomy. Studies by Doll and Hiraoka have shown that minimally invasive aortic valve surgery results in less postoperative drainage and less blood transfusion (13, 14). A large sample of minimally invasive AVR surgery (1,639 cases) reported by Gosev et al. showed that the 1, 5, 10, and 15-year survival rates after minimally invasive aortic valve replacement were 96, 93, 92, and 92%, indicating that minimally invasive aortic valve surgery has an excellent long-term survival rate (15).

In the past 10 years, with the rapid development of surgical techniques and operating instruments, endoscopy has been widely used in cardiac surgery. Total endoscopy has been commonly used in ASD repair, VSD repair, mitral valvuloplasty or replacement, tricuspid valvuloplasty or replacement, radiofrequency ablation of atrial fibrillation, myoma resection, and even coronary artery bypass grafting. However, due to the particularity of the anatomical structure of the aortic root, the space of the aortic root is small, and the operation is complex, which affects the development of total endoscopic techniques in AVR surgery. Vola M. et al. reported the world's first case of TE-AVR in 2014, and in 2016, they followed up with 14 low-risk patients who underwent TE-AVR and obtained inspiring results (16, 17). Vola adopted the sutureless prosthesis, which was proven to be an ideal prosthesis for TE-AVR for its convenient implantation. However, the sutureless prosthesis is not available in certain countries like China and is inadequate for young patients who should be implanted with mechanical valves. Combining foreign endoscopic aortic valve replacement technology and rich experience in valve angioplasty and replacement, the authors' center began to try TE-AVR in 2020. The authors' center uses the previous experience of total endoscopic mitral valve replacement surgery and adopts the “three-ports method,” but each working port is moved upward by one ICS. The main working port is in the 3rd ICS. Its specific location varies according to the aortic valve disease: in patients with aortic valve calcification and stenosis, the main working port can be located between the midclavicular line and the parasternal line, which is convenient for removal of calcified valves; in patients with aortic insufficiency, the main working port can be located on the lateral side of the midclavicular line, between the midclavicular line and the anterior axillary line, which is conducive to the exposure and administration of cardioplegia to the left and right coronary ostia. The main advantage of the “three-ports method” is that the left and right hands are flexible and not affected by the position of the ascending aorta.

Vola et al. used sutureless prostheses (Medtronic 3f Enable SU valve) that required no suturing and knotting to facilitate minimally invasive AVR, which decreased CPB and ACC times dramatically (18). However, sutureless prostheses are not available in some regions. The long-term durability data is still lacking. And the demands of mechanical valves for young patients should not be ignored. Thus, we started our practices in TE-AVR using standard surgical prostheses in 2020, including 20 mechanical and nine biological valves. The average CPB time and ACC time were (177.7 ± 43.2) min and (118.3 ± 29.7) min, respectively, but the mean CPB time and ACC time reported in Vola M et al. were (161 ± 31) min and (112 ± 18) min, respectively, which suggested using standard prostheses did not extent CPB and ACC times significantly by adopting proper endoscopic techniques. The types of prostheses used by all the patients in our study included a variety of prostheses commonly used at present, which also suggests that the type of prosthesis does not limit the TE-AVR. Implanting the prosthesis was more difficult than the mechanical valve in the actual operation process, which was mainly reflected in the knotting procession. Although the frame of bioprosthesis may affect knotting, especially in the right/left and right/non-commissural, it did not significantly prolong the operation time. Automated fastener devices or self-suturing devices, such as Cor-knot, helps simplify the knotting process, reducing knotting time while maintaining a tight knot. The Cor-knot was introduced into our center recently, and we used this device in two total endoscopic mitral repair surgeries. We are very impressed that this device is convenient and time-saving, and we plan to further use it in TE-AVR surgery. It is difficult to knot at the non-coronary sinus because of the limited space between the aortic wall and prosthesis valve frame for a minimally invasive knot pusher. Due to the unique design of the Cor-knot device, the thin tip of the Cor-knot device is directly pushed onto the sewing ring during the knotting process, which will make the knotting process fast, accurate, and reliable.

In the results of our study, although total endoscopic surgery prolonged cardiopulmonary bypass time and aortic occlusion time, it did not increase complications. At the same time, the mechanical ventilation time and postoperative hospital stay in the TE-AVR group were significantly shorter than those in the traditional thoracotomy group, indicating that the endoscopic group recovered faster. In addition, although there was no statistical difference in ICU stay and thoracic drainage volume, the TE-AVR group also showed a decreasing trend, indicating that the TE-AVR group had less surgical trauma, less bleeding, and faster recovery. This is consistent with the research results of minimally invasive aortic valve surgeries. Several studies have shown that minimally invasive aortic valve surgery has a lower incidence of postoperative atrial fibrillation than traditional surgery. However, this finding was not found in this study, which may be related to the relatively small sample size.

Van Praet et al. believed that the positional relationship between the ascending aorta and the sternum was significant for aortic surgery *via* parasternal transverse incision, and preoperative CT angiography of the aorta was essential to clarify the positional relationship between the ascending aorta and the sternum (19, 20). However, the position of the ascending aorta is not the limit of total endoscopic aortic valve surgery. Even if more than 50% of the ascending aorta is located on the left side of the sternum, it is still possible to use the minimally invasive method of total endoscopic surgery. In other words, total endoscopic surgery requires a certain distance between the operating point and the position of the endoscope, which is more convenient for surgical operations. In addition, the size of the aortic root and ascending aorta may be more critical factors affecting the process. The aortic root's limited operating space makes it difficult to seat the prosthesis valve even under direct vision. Therefore, the ascending aorta and aortic root should not be too small in patients undergoing TE-AVR. According to the authors' experience, caution should be exercised in patients with aortic root diameters < 25 mm. The adequate size of the aortic root is a favorable factor for completing the operation. Of course, the diameter of the aorta should be a manageable size. Otherwise, the aorta might not be fully cross-clamped. Severe leaflet and annular calcification will significantly increase the difficulty of minimally invasive aortic valve replacement. Therefore, we suggest choosing patients with aortic insufficiency or mild stenosis in the early learning curve stage.

## 5. Conclusion

The early clinical efficacy of total endoscopic aortic valve replacement is satisfactory, safe, and feasible. Compared with the traditional full sternotomy approach, the endoscopic surgical preserves the integrity of the sternum, has less bleeding, faster recovery, and improves patients' quality of life.

## Data availability statement

The raw data supporting the conclusions of this article will be made available by the authors, without undue reservation.

## Ethics statement

The studies involving human participants were reviewed and approved by Ethics Review Committee of Guangdong Provincial People's Hospital. Written informed consent for participation was not required for this study in accordance with the national legislation and the institutional requirements. Written informed consent was obtained from the individual(s) for the publication of any potentially identifiable images or data included in this article.

## Author contributions

WG, KZ, HmG, and HH contributed to the conception and design of the study. WG and ZW wrote the manuscript. All authors contributed to the manuscript revision and approved the submitted version.
